# Saturation magnetisation as an indicator of the disintegration of barium hexaferrite nanoplatelets during the surface functionalisation

**DOI:** 10.1038/s41598-023-28431-4

**Published:** 2023-01-19

**Authors:** Darja Lisjak, Iztok Arčon, Matic Poberžnik, Gabriela Herrero-Saboya, Ali Tufani, Andraž Mavrič, Matjaz Valant, Patricija Hribar Boštjančič, Alenka Mertelj, Darko Makovec, Layla Martin-Samos

**Affiliations:** 1 Jožef Stefan Institute, 1000 Ljubljana, Slovenia; 2 University of Nova Gorica, 5000 Nova Gorica, Slovenia; 3 CNR-IOM, Democritos National Simulation Center, Istituto Officina dei Materiali, c/o SiSSA, 34136 Trieste, Italy; 4 Jožef Stefan International Postgraduate School, 1000 Ljubljana, Slovenia

**Keywords:** Nanoscale materials, Materials chemistry

## Abstract

Barium hexaferrite nanoplatelets (BHF NPLs) are permanent nanomagnets with the magnetic easy axis aligned perpendicular to their basal plane. By combining this specific property with optimised surface chemistry, novel functional materials were developed, e.g., ferromagnetic ferrofluids and porous nanomagnets. We compared the interaction of chemically different phosphonic acids, hydrophobic and hydrophilic with 1–4 phosphonic groups, with BHF NPLs. A decrease in the saturation magnetisation after functionalising the BHF NPLs was correlated with the mass fraction of the nonmagnetic coating, whereas the saturation magnetisation of the NPLs coated with a tetraphosphonic acid at 80 °C was significantly lower than expected. We showed that such a substantial decrease in the saturation magnetisation originates from the disintegration of BHF NPLs, which was observed with atomic-resolution scanning transmission electron microscopy and confirmed by a computational study based on state-of-the-art first-principles calculations. Fe K-edge XANES (X-ray absorption near-edge structure) and EXAFS (Extended X-ray absorption fine structure) combined with Fourier-transformed infrared (FTIR) spectroscopy confirmed the formation of an Fe–phosphonate complex on the partly decomposed NPLs. Comparing our results with other functionalised magnetic nanoparticles confirmed that saturation magnetisation can be exploited to identify the disintegration of magnetic nanoparticles when insoluble disintegration products are formed.

## Introduction

Magnetic nanoparticles are of high scientific interest due to the nanosize effect on their properties, making them suitable for various applications, from techniques to medicine^1–5^. Most of these applications use superparamagnetic spinel ferrite (i.e., magnetite or maghemite) nanoparticles^6–8^. While, new exciting functional materials, such as ferromagnetic fluids, anisotropic magneto-optic composites, porous nanomagnets, spin-memory devices, and contrast agents for bioimaging, are based on barium hexaferrite nanoplatelets^9–14^. Such versatile applications require specific surface functionalisation (as example see Ref.^15^).

Functional ligands can attach to nanoparticles surfaces via an anchoring group. The coordination interaction between the surface metal ions and anchoring group depends on the system's properties (e.g., pH, temperature, solvent) because there is a balance between different interactions: surface metal–anchor–ligand, surface metal–solvent, ligand–solvent. In general, a strong coordinative interaction metal–anchor should prevail, but when the surface metal–anchor–ligand interaction is stronger than the surface metal–crystal interaction, the material disintegrates^12,16–19^. The process is similar to the dissolution of an ionic crystal where the stronger interaction metal–solvent prevails over the metal–crystal interaction. Therefore, "dissolution" is often used in literature instead of "disintegration".

In general, one could follow the dissolution and disintegration of a solid by chemical analyses of dissolved metals. However, such analysis is not straightforward when the disintegration products are in a solid state. A combination of several techniques, including surface-sensitive and spectroscopic techniques, and electron microscopy combined with microanalytics, should be applied. Each of the techniques requires a highly trained expert, most of them include expensive equipment and limited statistical/quantification options.

**Figure 1:**
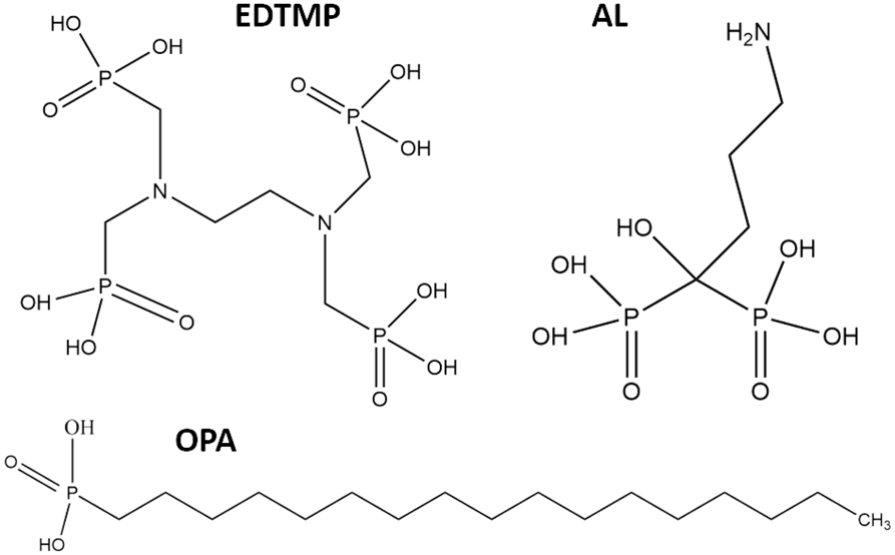
Chemical structures of ethylenediaminetetra (methylene phosphonic acid) (EDTMP), (4-amino-1-hydroxy-1-phosphonobutyl)phosphonic acid, also known as alendronic acid (AL), and octadecylphosphonic acid (OPA).

In this work, we propose exploiting saturation magnetisation as an indication of the potential disintegration of magnetic nanoparticles during their surface functionalisation. As a proof-of-concept, we focused first on ferrimagnetic barium hexaferrite nanoplatelets (BHF NPLs), as they have a well-defined crystal structure^20^. We functionalised the NPLs with different phosphonic acids (Fig. 1) because they ensure one of the strongest interaction with metal (oxide) surface via different bonding modes^21^. Finally, we discuss our results with those obtained on other functionalised magnetic nanoparticles.

The BHF crystal unit cell (see Fig. 7 in “Results and discussion”) is defined by two structural blocks, S and R, in an RSR*S* sequence along the *c*-axis^22,23^. The S block ((Fe_6_O_8_)^2+^) represents a slice of the cubic spinel structure, the R block ((BaFe_6_O_11_)^2−^) is hexagonal, and * denotes the rotation of the block for 180° around the *c*-axis. Fe^3+^ ions occupy five crystal lattice sites: an octahedral (*2a*) and tetrahedral (*4f*_*1*_) in the S block, an octahedral (*4f*_*2*_) and bipyramidal (*2b*) in the R block, and an octahedral (*12k*) site at the S–R interface. As shown recently, the hydrothermally synthesised BHF NPLs terminate with the S block, in particular with the Fe^3+^ at *12k* sites, just below the oxygen ions at the very surface^20^. This structural deviation of BHF NPLs vs bulk, i.e., (RS)_n_S vs (RS)_2_, also results in different compositions of the BHF NPLs (e.g., BaFe_15_O_23_ for n = 2) vs bulk BHF (BaFe_12_O_19_). Hexaferrites grow preferentially in the *ab* crystal plane into thin platelets with the magnetic easy axis aligned perpendicular to the *ab* plane, i.e., in the *c*-direction. Therefore, any change of the terminal crystal plane should affect their saturation magnetisation.

The room-temperature saturation magnetisation given per mass of the sample (Ms) decreased after functionalising the NPLs with any of the selected phosphonic acids. The Ms reduction was correlated with the mass fraction of the nonmagnetic surface coating, except for the ethylenediaminetetra (methylene phosphonic acid) (EDTMP) coating with around 10–20% larger decrease in the Ms than predicted. Detailed spectroscopic analyses suggested partial disintegration of the NPLs by EDTMP, which was demonstrated by Fe K-edge X-ray absorption near-edge structure (XANES) and atomically-resolved scanning transmission electron microscopy (ARM-STEM). Furthermore, the first-principle calculations excluded an effect of the surface metal–anchor bond on the Ms of NPLs but was attributed to the crystal surface termination. The difference between the measured Ms and the Ms normalised to the surface-ligand mass fraction is directly correlated to the disintegration of magnetic nanoparticles during the functionalisation.

## Results and discussion

### BSHF NPLs coated with EDTMP or OPA

**Figure 2:**
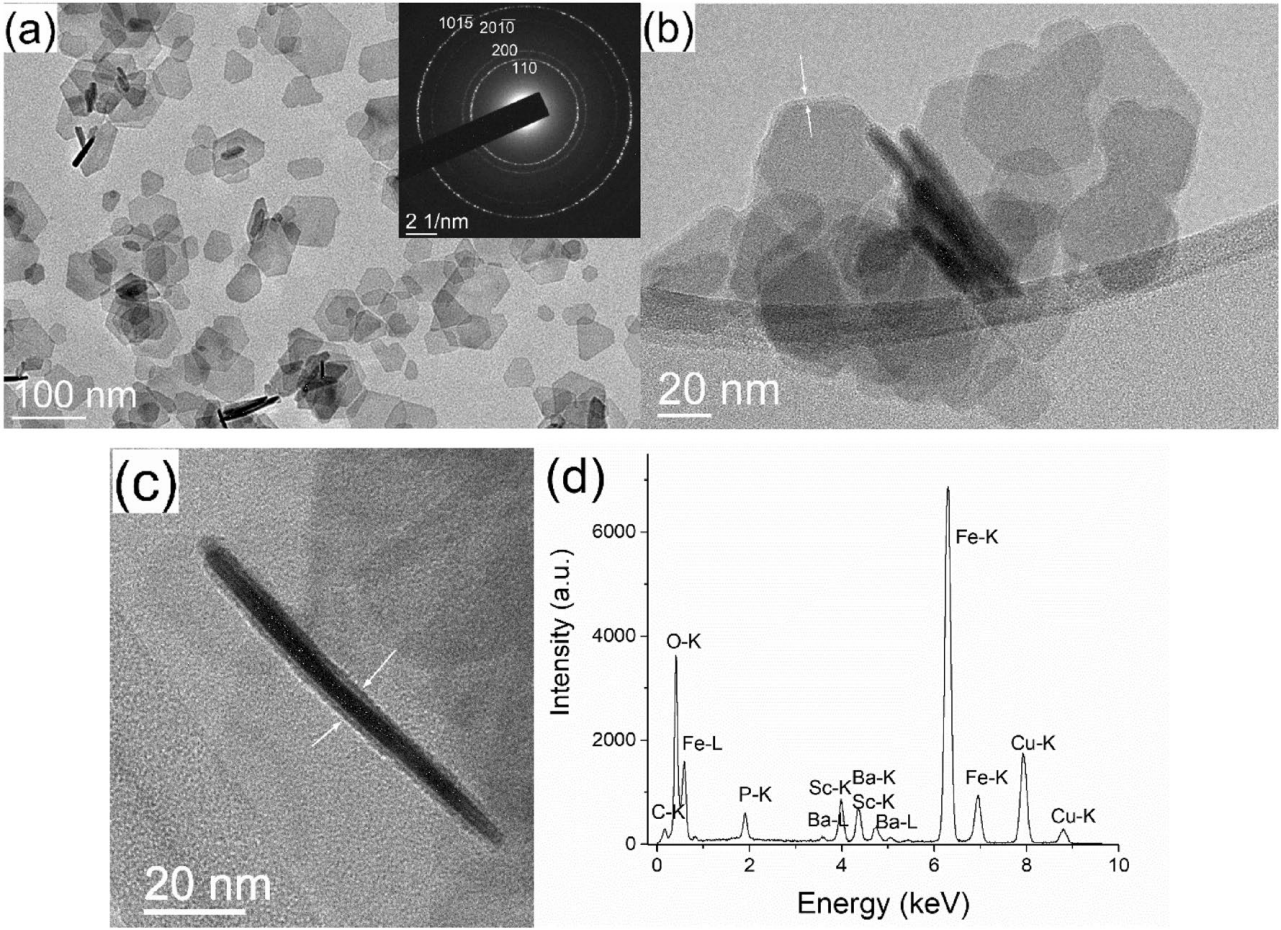
TEM images of the (**a**) as-synthesized BSHF NPLs with the corresponding SAED, (**b**) BSHF NPLs coated at RT with OPA and (**c**) EDTMP, and (**d**) the EDXS spectrum of BSHF NPLs@EDTMP. The indices in SAED correspond to the hexagonal space group P6_3_/mmc (194). The arrows in panels (**b**) and (**c**) point at the amorphous surface layer.

**Figure 3:**
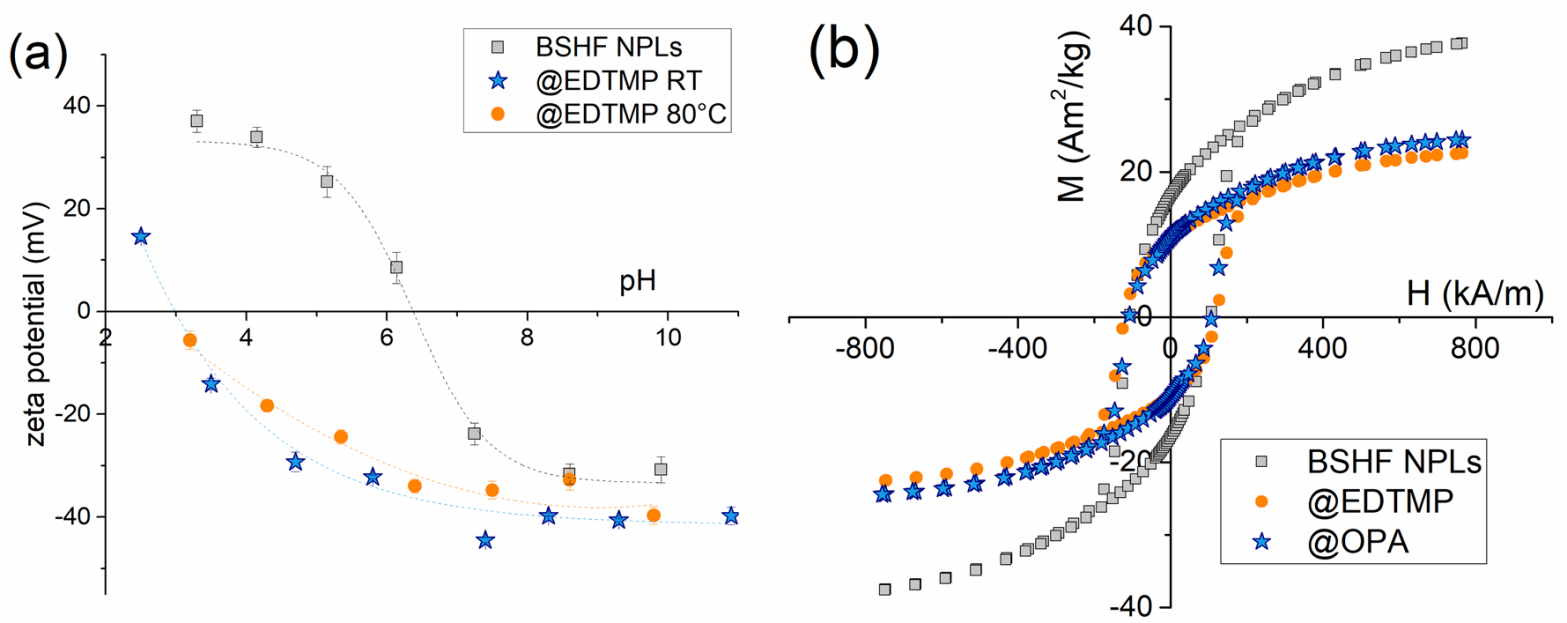
(**a**) Zeta-potential behaviour of the BSHF NPLs aqueous suspensions, and (**b**) magnetic hysteresis loops of the core and coated BSHF NPLs. The Ms values are given in Table 1. The lines in panel (**a**) are a guide to the eye.

Barium hexaferrite NPLs were partly substituted with Sc^3+^ to narrow their size distribution while keeping applicable magnetic properties^24^. The partial Sc^3+^-substitution for Fe^3+^ does not affect the structure and composition of the surface crystal planes^25^. Consequently, the interaction with the surface ligands should be the same for the pure (BHF) or Sc-substituted (BSHF) NPLs. BSHF NPLs were synthesised in three batches. The as-synthesised BSHF NPLs from all batches were in the form of thin NPLs with a mean diameter of ~ 50 nm and thickness of 3–4 nm (Fig. 2a). Selected area electron diffraction (SAED) confirmed the magnetoplumbite structure of NPLs. All NPLs showed hard-magnetic properties with a coercivity (Hc) of ~ 79.6 kA/m and saturation magnetisation Ms > 30 Am^2^/kg (Table 1, also see Fig. 3b).

**Table 1:** Mass fraction of the coated ligand (ω), room-temperature Ms values (i.e., the magnetization at the magnetic field of 1 T = 796 kA/m) of the core and coated particles, and the predicted Ms = $\left(1-\omega \right){Ms}_{core}$ values of the coated NPLs.

Sample	Ligand fraction (wt%)	Measured Ms (Am^2^/kg)	Predicted Ms (Am^2^/kg)	Data origin
BSHF NPLs^a^, 49 (20) nm	/	34.6 ± 0.3		This study
BSHF NPLs^b^ 48 (24) nm	/	35.4 ± 0.4	/	Previous study
BSHF NPLs^c^ 60 (25) nm	/	37.7 ± 0.4	/	Previous study
BSHF@EDTMP-8^a^ RT	11.7	29.9 ± 0.3	30.6	This study
BSHF@EDTMP-8^b^ 80 °C	17.0	25.9 ± 0.5	29.4	Previous study
	18.0	22.6 ± 0.2	29.0	This study
BSHF@EDTMP-8^c^ 80 °C	17.8	26.0 ± 0.3	31.0	This study
BSHF@OPA-17^b^ RT	35.9	24.4 ± 0.2	22.7	Previous study
BSHF@DPA-8^b^ 80 °C	29.6	23.0 ± 0.2	24.9	Previous study
BSHF@PSA-20^b^ 80 °C	28.6	26.8 ± 0.3	25.3	Previous study
BSHF@AL-10^a^ 80 °C	12.0	30.3 ± 0.3	30.4	This study

The coated NPLs were prepared at RT or 80 °C. The listed data were obtained within this and the previous study^12^.

^a–c^Denote different batches of the core BSHF NPLs with a different mean diameter and standard deviation in brackets. Uncertainty of the Ms values originates from the limited precision of the balance (± 1%).

The EDTMP and octadecylphosphonic acid (OPA) coatings were observed as thin (1–2 nm) amorphous surface layers on BSHF NPLs (see examples in Fig. 2b,c). In addition to the constituent BSHF elements (i.e., Ba, Fe, Sc, and O), P was also detected in all coated NPLs with energy dispersive X-ray spectroscopy (EDXS), confirming that the coatings were of the phosphonate origin. An example is shown for the BSHF@EDTMP NPLs in Fig. 2d. The Cu and C peaks originate from the TEM supporting grid, but the C signal also comes from EDTMP.

BSHF@OPA NPLs in toluene suspensions were hydrophobic. In contrast, BSHF@EDTMP NPLs formed stable aqueous suspensions. Highly negative zeta-potential values (Fig. 3a) suggested a strong electrostatic repulsion between BSHF@EDTMP NPLs reflected in their high colloidal stability. No significant difference was observed if the EDTMP coating was obtained at room temperature (RT) or 80 °C suggesting a similar density of acidic surface groups.

The coatings did not affect the Hc values of BSHF NPLs. Only a decrease in Ms was observed (Fig. 3b, Table 1) because of the nonmagnetic-coating contribution to the overall sample's mass. The observed decrease roughly agrees with the predicted Ms values calculated using the surface-ligand mass fraction determined with thermogravimetric analysis (TGA). However, the Ms value of the BSHF@EDTMP obtained at 80 °C decreased much more (12–22%) than expected. The unexpected decrease was confirmed for several batches and cannot be attributed to the potential error related to the measurement uncertainty. Such a substantial decrease in the Ms values indicates changes in the magnetic structure after the coating process.

**Figure 4:**
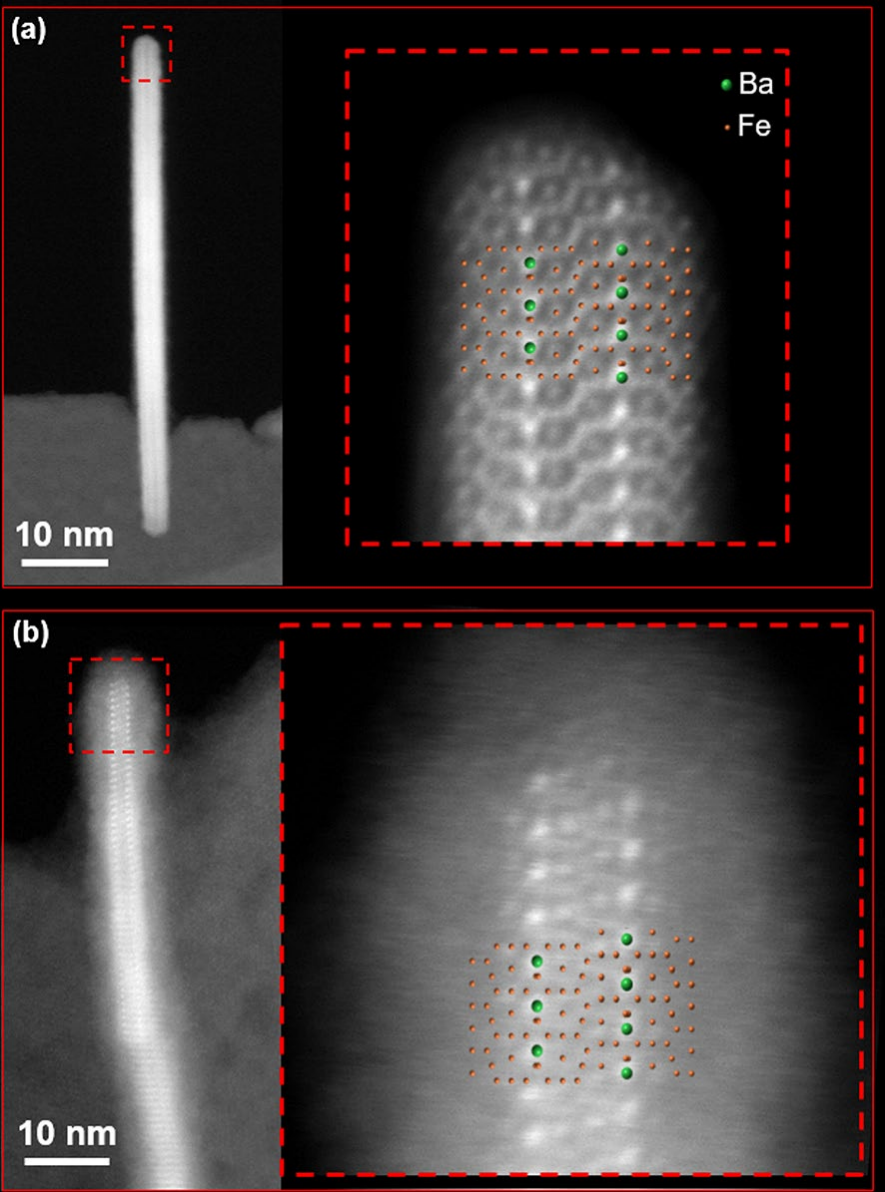
(**a**) HAADF STEM image of the bare BSHF NPL, and **(b)** BSHF@EDTMP NPL. The NPLs are oriented edge-on, with the hexaferrite structure oriented along <10–10> direction. The projected structural model is superimposed over the enlarged images to illustrate the positions of the Ba^2+^ (green) and Fe^3+^ (orange) ions across the complete NPL structure.

Partial decomposition of the BSHF NPLs observed with ARM-STEM can alter the magnetic-exchange interactions and consequently also the Ms values. An example of such disintegration is shown in Fig. 4. The outmost surface crystal planes of the S block are missing on a BSHF@EDTMP NPL (Fig. 4b) in comparison to the as-synthesised BSHF NPL for which the surface S-block is complete (Fig. 4a). No evidence of any decomposition was found after coating the NPLs with OPA, which is in accordance with the Ms measurements.

### Spectroscopic analyses of the BHSF NPLs

A combination of spectroscopic analyses was performed on the EDTMP- and OPA-coated BSHF NPLs and compared with the core NPLs and reference Fe-complexes (Fe–EDTMP and Fe–OPA). We considered the BSHF@EDTMP and reference Fe–EDTMP samples prepared at 80 °C, and BSHF@OPA and reference Fe–OPA samples prepared at RT.

Some structural changes of the EDTMP and OPA phosphonic groups, when coated onto BSHF NPLs, were assumed when comparing the attenuated total reflectance Fourier transform infrared (ATR-FTIR) spectra of pure acids with the coated samples (Fig. S1). As also shown previously^12,26,27^, typical bands corresponding to the phosphonic groups in EDTMP and OPA merged into a broad band after being coated onto a metal-oxide surface. No distinct difference was observed between differently coated BSHF NPLs. On the contrary, the ATR-FTIR spectra of BSHF@EDTMP NPLs and the reference Fe–EDTMP complex (Fig. S1) are significantly different, with the latter resembling more to the pure EDTMP than to the BSHF@EDTMP NPLs. However, an intense broad band at ~ 1078 cm^–1^ in the Fe–EDTMP spectrum (not observed for the pure EDTMP) can be associated with the phosphonate structure, similar to the one in BSHF@EDTMP.

**Figure 5:**
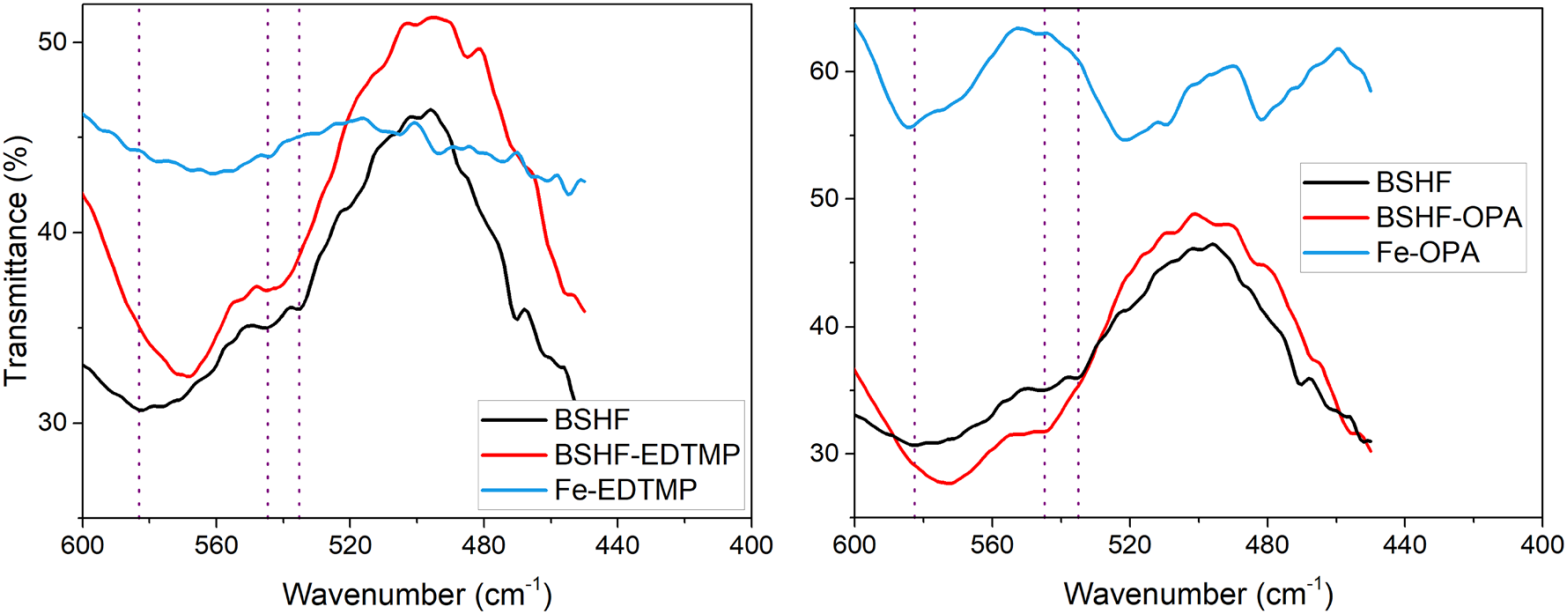
DRIFT spectra of the core and coated BSHF NPLs and of the Fe-complexes. The dotted lines represent typical Fe–O vibration bands in the core BSHF NPLs.

We used diffusion reflectance infrared Fourier transform (DRIFT) spectroscopy, allowing us to measure at lower wavenumbers (< 600 cm^–1^) that are typical for the Fe–O vibrations (Fig. 5). A redshift of the Fe–O vibrations at 580 cm^–1^ with respect to the core BSHF NPLs^28–30^ can be observed and suggests the coordination interaction between BSHF and EDTMP/OPA coatings^31,32^. In the DRIFT spectrum of the reference Fe–EDTMP complex, it is very difficult to distinguish the typical Fe–O bands as opposed to the BSHF@EDTMP spectrum. In contrast, the DRIFT spectrum of the reference Fe–OPA complex clearly shows a blueshift of Fe–O band observed at 580 cm^–1^ for the core BSHF NPLs, which is the opposite than observed for the BSHF@OPA NPLs. These results suggest some similar structural features related to the Fe–O–P in the coated BSHF NPLs and Fe–EDTMP complex, whereas the Fe–O–P structure is different in the Fe–OPA complex.

**Figure 6:**
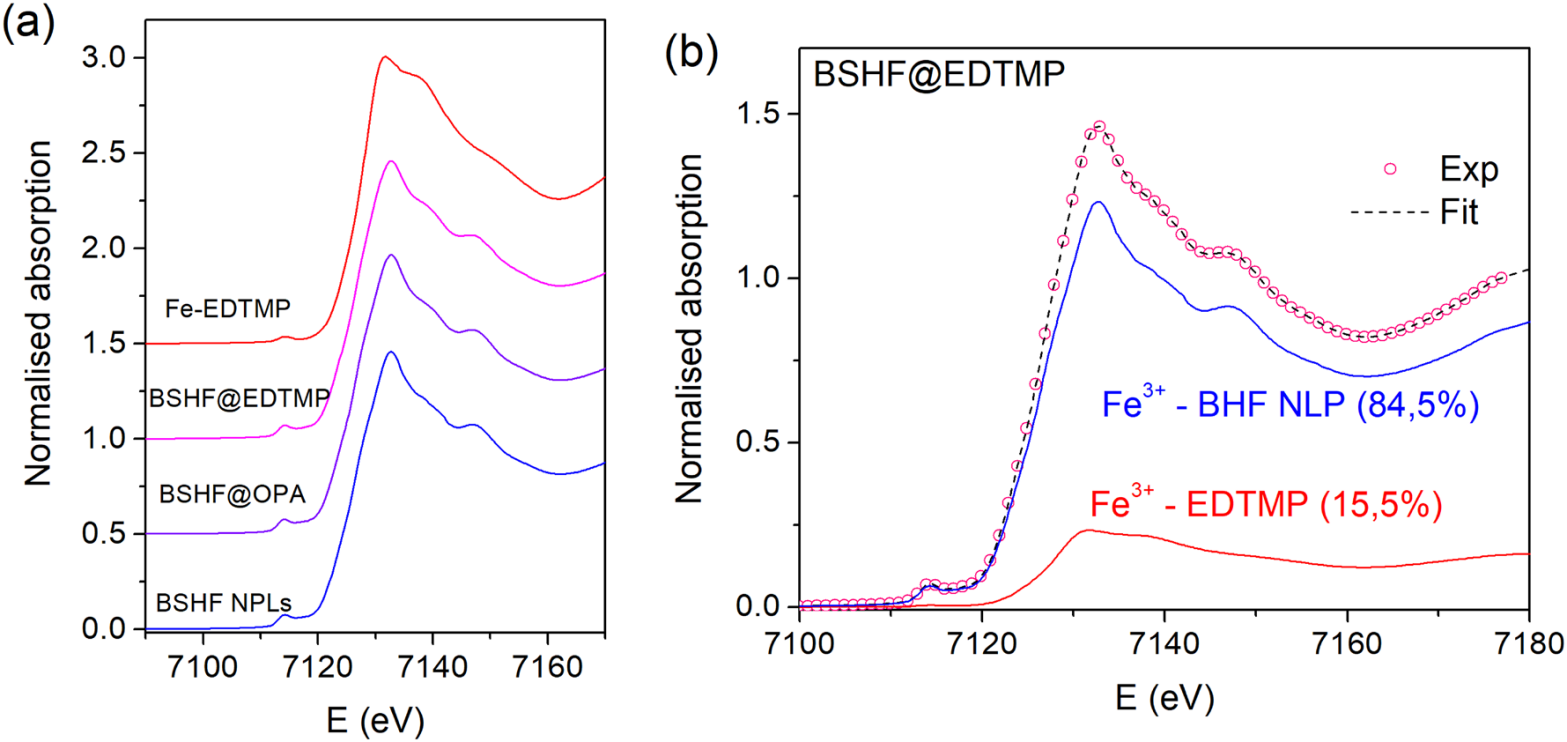
(**a**) Fe K-edge XANES spectra of the EDTMP and OPA coated BSHF NPLs and the reference spectra of uncoated BSHF NPL sample and Fe–EDTMP complex. The spectra are shifted vertically for clarity. (**b**) LCF analysis of the BSHF@EDTMP spectrum: red circles—experiment; blue dashed line—best fit with a linear combination of reference XANES profiles of BSHF NPLs (84.5%) and Fe–EDTMP complex (15.5%), plotted below.

The Fe K-edge XANES analysis was applied to examine Fe cations' valence and local symmetry in the EDTMP- and OPA-coated BHF NPL samples. The normalized Fe K-edge XANES spectra are shown in Fig. 6 together with the spectra of the corresponding Fe^3+^ reference compounds (core BSHF NPLs and Fe–EDTMP complex). Different local symmetries and environments of Fe cations result in different K-edge profiles in the XANES spectra, and the energy position of the Fe K-edge is correlated with the valence state of the Fe cations in the sample. The Fe K-edge shift of about 4.5 eV was found between the spectra of the di- and trivalent compounds^33,34^. The Fe K-edge energy position in the BSHF@EDTMP and BSHF@OPA NPLs spectra is the same as in the spectra of the Fe^3+^ reference compounds, showing that Fe cations in both samples are in trivalent form. The Fe K-edge profiles of the two samples are similar to that of the BSHF NPL reference sample. However, a principal component analysis (PCA) indicates small differences between the spectra of the two samples and the BSHF NPL reference. A linear combination fit (LCF) analysis shows that XANES spectra of both samples can be completely described by a linear combination of the two reference XANES profiles: BSHF NPLs and Fe–EDTMP complex. The result of the LCF analysis is illustrated in Fig. 6b. The LCF results indicate that both samples are mixtures of two phases, one with Fe cations in the BSHF NPLs and the other with Fe cations coordinated to phosphonic groups (i.e., as Fe–EDTMP or Fe–OPA complex). In the BSHF@EDTMP NPLs, 84.5% Fe cations are incorporated in the BSHF NPLs, and 15.5% are in the form of Fe–EDTMP complex, while in BSHF@OPA NPLs, only a negligible amount (below 0.5%) of Fe cations is coordinated to phosphonic groups in the Fe–OPA complex, and the majority (99.5%) are incorporated in the BSHF NPLs. Uncertainty of relative amounts of the two species in the sample, as estimated by the LCF analysis, is ± 0.1%.

Overall, the XANES results confirm different structures of EDTMP vs OPA coatings with the BSHF NPLs. The results of the LCF analysis of BSHF@EDTMP demonstrate the partial decomposition of BSHF NPLs when coated with EDTMP at 80 °C. 15% of Fe cations are extracted from the crystal structure of BSHF NPLs, and the extracted Fe cations interact with phosphonic groups, forming Fe–EDTMP complexes within the (1–2 nm) amorphous phosphonate coating on the BSHF NPLs. The result agrees with EDXS^12^ and DRIFT (Fig. 5) analyses. Such decomposition is negligible in the BSHF@OPA NPLs and was not observed with ARM-STEM nor detected with the EDXS.

The Fe K-edge X-ray absorption fine structure (EXAFS) analysis was used to detect the average local structure of Fe cations in the EDTMP- and OPA-coated BSHF NPLs. The details of EXAFS analysis are presented in the Supplementary Information (Fig. S2 and Tables S1–S4). The Fe K-edge EXAFS results show that the average Fe local structure in both samples (BSHF@EDTMP and BSHF@OPA NPLs) is practically the same as in the BSHF NPL reference sample. The signal from the small fraction of Fe cations bound to the phosphonic groups in the coatings, demonstrated by XANES analysis, is not excluded by EXAFS fits; however, their contribution to the total EXAFS signal is below the detection limit. Also, the signal of the adsorbed phosphonate ligands to the surface Fe cations of BHF NPLs, which may be present in a relatively small relative amount in these samples, is below the detection limit of EXAFS analysis.

### First-principles computational modelling

**Figure 7:**
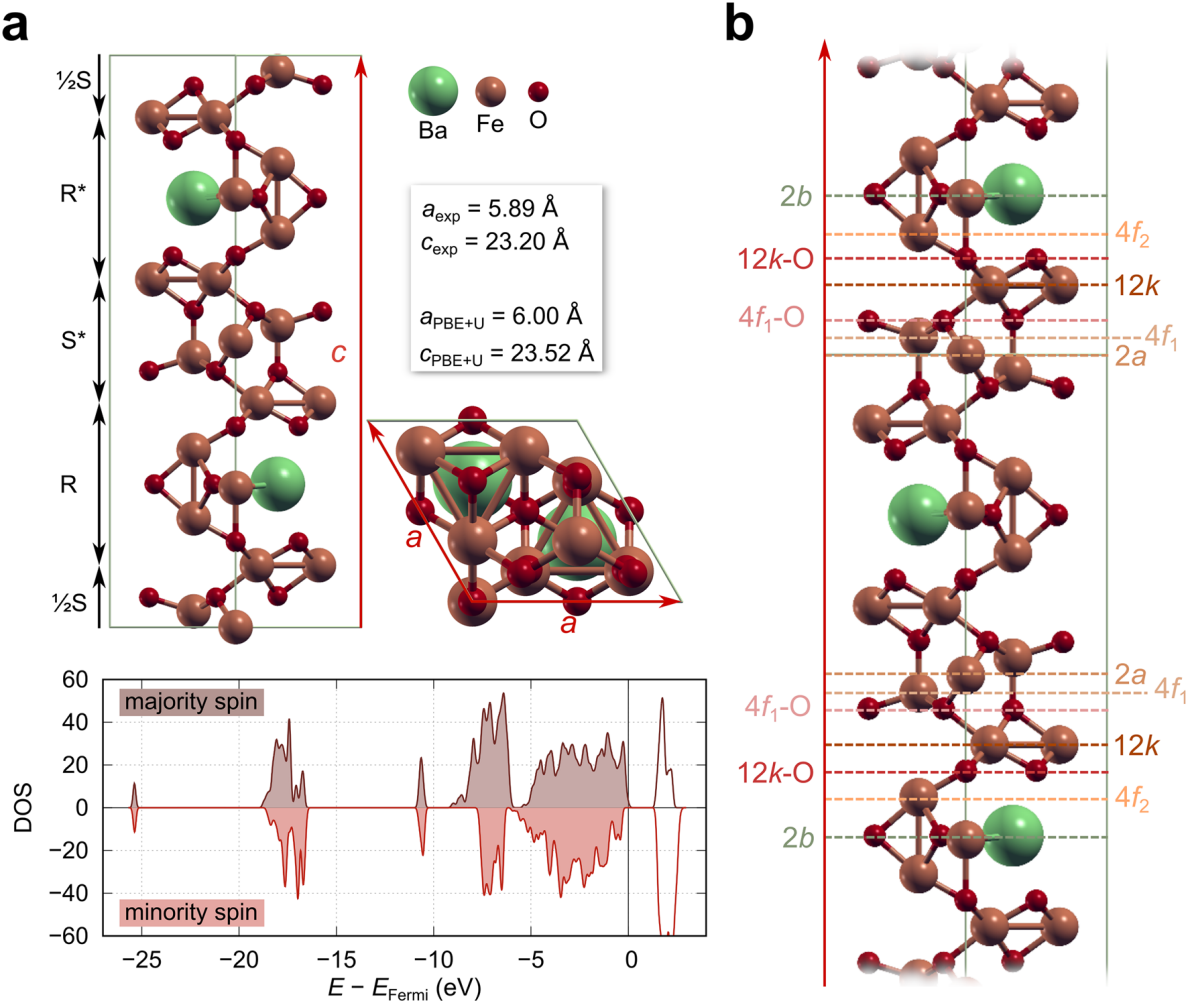
BHF-based systems (**a**) The top part shows the bulk structure of BHF and a comparison of experimental lattice parameters to those obtained with DFT + U. The bottom part shows the energy DOS obtained with spin-resolved DFT + U calculations. The two spin projections are represented in brown (majority spin) and red (minority spin), respectively and the occupied states are filled. The estimated bandgap is 1.4 eV, in line with the measured optical adsorption edge of 1.82–1.86 eV^38^. (**b**) The different bulk terminations of BHF bulk and their designations. The figure was generated by using inkscape 1.2.1 (https://inkscape.org/), gimp 2.10 (https://www.gimp.org/), gnuplot 5.4 (http://www.gnuplot.info/), and xcrysden 1.6.2^41^.

The primary goal of our computational modelling was to elucidate the effect of the adsorbed phosphonate-based ligands on the magnetic properties of the BHF NPLs. In order to build a parameter-free model of the BHF NPLs, all calculations were performed within the framework of Density Functional Theory corrected by a Hubbard term^35^ for which U was determined self-consistently^36^ (average U value = 4.3 eV for BHF bulk). The BHF bulk optimised lattice parameters *a* = 6.00 Å and *c* = 23.52 Å (Fig. 7a), are in close agreement with their experimental values *a* = 5.89 Å and *c* = 23.20 Å ^37^. Regarding its electronic structure, we obtained an electrical gap of BHF bulk of 1.4 eV, as represented in the energy Density of States (DOS) in Fig. 7a. This value is in line with the measured optical adsorption edge of 1.82–1.86 eV^38^. The calculated total magnetisation of 40 μ_bohr_ per unit cell is consistent with a contribution of 5 μ_bohr_ per Fe^3+^ ion and the existence of two antiferromagnetic sublattices (lattice sites *2a*, *2b* and *12k* versus *4f1* and *4f2*). Note that a magnetic moment of 5 μ_bohr_ per Fe^3+^ ion is compliant with the maximal spin multiplicity of an isolated Fe^3+^ ion. Our calculations confirm that the difference between electronic spin densities is mainly located in Fe^3+^ ions, whereas the other atomic species do not contribute significantly. Consequently, magnetic properties are dictated by the highly localised d electrons. The estimated magnetic moment is also in line with previous first-principles calculations^39,40^ and the measured magnetisation of 72 Am^2^/kg at room temperature^37^. The majority spin contributions are labelled as "spin up" throughout the text, whereas the minority is named "spin down".

As BHF NPLs present a relatively large surface area to thickness ratio, a single NPL was approximated as a slab subject to periodic boundary conditions (PBC) for the basal directions and a vacuum layer along the main axis, i.e. the *c*-axis. The iron-terminated surfaces are labelled according to the corresponding iron lattice sites (e.g., *12k*), whereas the oxygen-terminated surfaces are denoted by the closest iron layer (e.g., *12k-O*), see (Fig. 7b). For a more detailed description of the construction of different bulk terminations see Section S2 of the Supplementary Information.

In order to study the adsorption of phosphonates on the NPLs, we chose a slab with the bulk termination that better agrees with current experimental measurements and observations. From the ARM-STEM measurements, the *12k-O* termination was assigned to the surface morphology of the NPLs^20^. However, given the manufacturing process, one can assume that such a surface is most likely fully hydroxylated. The *12k-O* slab was therefore saturated with hydrogen atoms (forming hydroxyl groups) and used as a reference point for the adsorption calculations.

Additionally, it is convenient to divide the structure of organic ligands into two structural elements: (i) the anchor group, responsible for adhesion to the surface and (ii) the backbone, responsible for lateral interactions within the formed organic layer. As a first step in studying the adsorption of phosphonate-based ligands, we chose a model ligand (CH_3_PO_3_H_2_) which consists of the –PO_3_H_2_ anchor group and a minimal backbone in the form of a methyl group. Moreover, two adsorption modes were considered for the model ligand: (i) the plain adsorption mode, which does not involve a chemical reaction, leading to adsorption through the formation of hydrogen bonds and (ii) adsorption via condensation, which involves the reaction of an H atom of the ligand molecule with a surface OH group, resulting in the bonding of the molecule to a surface Fe^3+^ cation and a water molecule as a side product. The two modes correspond to the following chemical reactions:

1 $${\text{MolH }} + \, * \to {\text{MolH}}*$$

2 $${\text{MolH }} + {\text{ OH}}* \, \to {\text{ Mol}}* \, + {\text{ H}}_{{2}} {\text{O}}$$

where MolH is a generic label for the intact anchor group, Mol represents an anchor group without one H atom and * stands for a free surface adsorption site.

For the plain adsorption mode the reaction energy (*∆E*) is calculated as:

3 $$\Delta E \, = \, E_{MolH/slab} - \, E_{slab} - \, E_{MolH}$$

where *E*_*MolH/slab*_, *E*_*slab*_, and *E*_*MolH*_ are the calculated total energies of the ligand adsorbed on the slab, the standalone slab, and the intact ligand in the gas phase. For adsorption via condensation, the reaction energy is calculated as:

4 $$\Delta E \, = \, E_{Mol/slab} + \, E_{H2O} - \, E_{slab} - \, E_{MolH}$$

where *E*_*Mol/slab*_ is the total energy of the ligand adsorbed on the surface without one H atom, and *E*_*H2O*_ is the total energy of a water molecule in the gas phase, whereas the other terms are analogous to those in Eq. (1).

**Figure 8:**
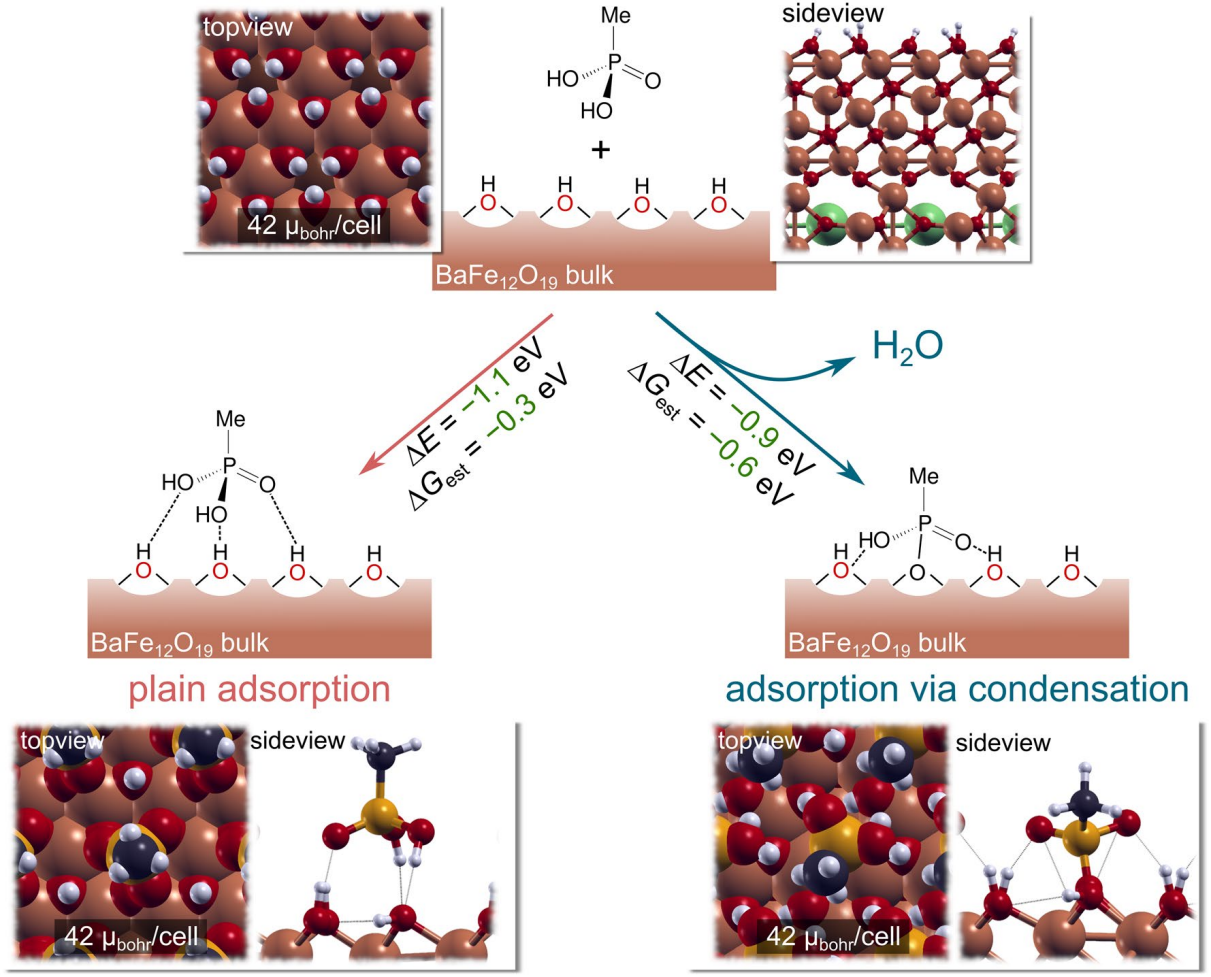
The reaction energy (*∆E*), estimated Gibbs free energy (*∆G*_*est*_) and computed total magnetization for the phosphonate adsorption in the plain mode (left) or via condensation (right). Both reaction energies are exothermic, indicating that phosphonates adsorb on the surface of BHF. The computed magnetization remains unchanged, irrespective of the mode considered. The figure was generated by using inkscape 1.2.1 (https://inkscape.org/), gimp 2.10 (https://www.gimp.org/), and xcrysden 1.6.2^41^.

The calculated reaction energies (*∆E*) for both mechanisms are shown in Fig. 8. Note that only the most stable configuration of the ligand in a (1 × 1) fully hydroxylated *12k-O* surface is shown for each adsorption mode. The *∆E* values for both considered modes are exothermic in the gas phase, with a value of − 1.1 eV and − 0.9 eV for plain adsorption and adsorption via condensation, respectively. From these *∆E* values, one could conclude that plain adsorption is slightly preferred over adsorption via condensation. However, it should be noted that a stronger bond (e.g., a covalent bond of ~ 5 eV) is formed through the latter mode. In fact, the corresponding *∆E* has a lower magnitude because it is a sum of multiple energetic contributions from both bond breaking and bond making. On the other hand, through the plain adsorption mode, bonds with lower binding energies in the range of ~ 0.1–1 eV are obtained.

In addition to this consideration, we remark that the reported values do not consider the change in entropy due to the immobilization of the ligand. The full calculation of Gibbs free energies is computationally very expensive and not feasible for the considered systems. However, as was shown in a previous publication^42^, the main entropic contribution stems from the loss of rotational and translational degrees of freedom as the molecule adsorbs on the surface. These values can be calculated for the molecule in the gas phase, and for the CH_3_PO_3_H_2_ ligand, the *TS*_*rot*+*tr*_ is equal to − 0.85 eV, at *T* = 289 K and *p* = 1 atm. Thus, the estimated Gibbs free energy (*∆G*_*est*_ = *∆E − T∆S*_*rot*+*tr*_) would be equal to − 0.25 eV for the plain adsorption mode. On the other hand, a water molecule is formed as a side product for adsorption via condensation, and the loss of the roto + translational degrees of freedom is partially counteracted. The *TS*_*rot*+*tr*_ for a water molecule in the gas phase equals − 0.58 eV, at *T* = 289 K and *p* = 1 atm. Given this counteracting effect, the estimated *∆G*_*es*t_ for adsorption via condensation is − 0.6 eV, indicating that this mode is more favourable than plain adsorption in the gas phase.

Our computational approach corroborated the lack of influence of the adsorbed phosphonate-based ligands on the magnetic moment of the BHF NPL (Section “BSHF NPLs coated with EDTMP or OPA”). As shown in Fig. 8, the net magnetic moment of the slab remains unchanged, regardless of the adsorption mode considered. More concretely, the calculated magnetic moment of a fully hydroxylated *12k-O* surface is 42 μ_bohr_/cell, remaining invariant for the two adsorption modes. According to our calculations, we can conclude that the experimentally observed changes in the Ms values for the BSHF coated with EDTMP at 80 °C do not stem from the adsorption of the ligand. This agrees with the observed negligible effect of the coatings on the average Fe local structure (Fig. S2 and Tables S1, S3 and S4).

An alternative explanation to the observed change in magnetic moments due to the adsorbed EDTMP ligand is the degradation of the BHSF NPLs, resulting in the dissolution of the surface crystal planes (as in Fig. 4b) or even an entire S block (as shown previously in^12^). In order to shed light on the matter, the thickness and surface morphology of the BHF NPLs were considered as potential causes for the observed changes. We cut slabs of different thicknesses from the BHF bulk (Fig. 7b) and calculated the net magnetization for each considered termination. Our results (Table 2) indicate a strong dependence of the magnetic moment of the slab on the specific bulk termination. The change in magnetization can be solely attributed to the number of Fe^3+^ ions in each of the antiferromagnetically aligned sublattices (Fe_up_ and Fe_down_ in Table 2), as evident by the expected versus computed magnetization values. For instance, the *4f2* terminated surface loses two Fe_up_ with respect to the *2b* termination, resulting in a magnetization decrease of 8 μ_bohr_/cell. From first-principles modelling, we conclude that the loss of magnetic moment in the case of the adsorption of EDTMP is not due to the adsorption of the ligand; instead, it is a consequence of the observed NPLs disintegration.

**Table 2:** Magnetic properties of the considered BHF bulk terminations are represented in Fig. 7b.

Surface termination	Fe_up_, Fe_down_	Expected magnetization^a^ (μ_bohr_/cell)	Computed magnetization (μ_bohr_/cell)	Computed magnetization (Am^2^/kg)
*2b*	17, 8	45	44.0	99.7
*4f*_*2*_	15, 8	35	36.0	96.1
*12k-*O	15, 6	45	40.0	112.7
*12k*	15, 6	45	41.1	131.8
*4f*_*1*_*-*O	9, 6	15	18.5	73.8
*4f*_*1*_	9, 6	15	15.4	67.3
*2a*	9, 4	25	24.0	114.8

The expected magnetization is simply estimated from the difference between the number of Fe_up_ and Fe_down_ ions, assuming a 3 + oxidation state. These values are compared to the computed magnetization.

^a^Note that the expected magnetization should not be mistaken with the predicted Ms values from Table 1.

### Comparison with other functionalised magnetic nanoparticles

Different interactions of EDTMP and OPA with the BSHF NPLs surfaces can be explained by their different chemical properties and structure. Any disintegration or dissolution of an ionic crystal, such as BSHF, is more probable in water than in less polar solvents. A water-soluble EDTMP with four phosphonic groups showed a much stronger interaction with BSHF NPLs than OPA, i.e., a monophosphonic acid, insoluble in water. In line with this, the magnetite structure and size remained intact when coating the nanoparticles with phosphonated stilbene in tetrahydrofuran^43^. Moreover, the resulting decrease of the Ms values corresponded to the adsorbed ligand fraction.

When considering aqueous systems, the interaction of phosphonic acids with the surface Fe^3+^ ions of BSHF NPLs should increase with an increasing number of the interacting phosphonic groups per ligand. Therefore, the second strongest interaction, after a tetraphosphonic acid (i.e., EDTMP), is expected from a water-soluble bisphosphonic ligand, such as alendronic acid (AL) (Fig. 1). To allow for a direct comparison with EDTMP, we coated the BSHF NPLs with AL under the same conditions, i.e., one deprotonated OH group in each phosphonic group (see experimental details in Supplementary Information, Section S1.2). The AL coatings formed on the BSHF NPLs surfaces at RT and 80 °C in water, similar to the EDTMP coatings. They were also observed as an amorphous surface layer with a thickness of 1–2 nm, and an EDXS analysis confirmed that the coatings were of phosphonate origin (Fig. S3). The surface-chemistry modification of NPLs by AL was evidenced from the zeta-potential behaviour versus pH (Fig. S4a). No significant difference was measured for the coatings of the same composition obtained at 80 °C and RT. The isoelectric point shifted from pH ~ 7 for core BSHF NPLs (Fig. 3a) to lower pH values after the coating, suggesting the presence of the surface acidic groups. This means that AL did not form a uniform monolayer on the NPLs' surfaces, similar to EDTMP. However, in contrast to the EDTMP coatings, the Ms values did not change more than expected after coating the NPLs with AL (Table 1 and Fig. S4b), suggesting an intact terminal crystal plane of the BSHF NPLs even when the coating was obtained at 80 °C. An important difference between the ligands is the distribution of all four phosphonic groups at different C atoms in EDTMP, whereas both phosphonic groups of AL are bonded with a single C atom. Consequently, different bonding modes of EDTMP and AL with BSHF NPLs can be assumed. In particular, a bidentate mononuclear bond favours the dissolution (correlated with EDTMP), and the bidentate binuclear mode inhibits the dissolution (correlated with AL)^44^.

In line with the above, a similar decomposition as in Fig. 4b was observed with ARM-STEM for the BSHF NPLs coated with a diphosphonic acid, i.e., (12-phosphono)dodecyl phosphonic acid (DPA; Fig. S5) and was accompanied by around 8% larger decrease in the Ms (Table 1) than predicted. In contrast to this, coating BSHF NPLs with a water-soluble monophosphonic acid, i.e., (12-phosphono)dodecyl sulfonic acid (PSA; Fig. S5), did not significantly decrease the Ms values (Table 1). We can conclude that the BSHF NPLs decompose at 80 °C in the aqueous solution of the phosphonic acids having at least two phosphonic groups at different C atoms, such as EDTMP and DPA.

Our spectroscopic studies (Figs. 4 and 5) show that a Fe–EDTMP coating formed at the NPLs surfaces. Such core–shell NPLs are composed of the magnetically ordered (i.e., ferrimagnetic) core and magnetically disordered (i.e., paramagnetic) shell. By combining the Ms values with the TGA data, one can quantify the degree of nanoparticles' disintegration as $\left[{Ms}_{predicted}-{Ms}_{measured}\right]/{Ms}_{predicted}$. In our case, the crystalline order in BSHF NPLs decreased by around 15 wt.% when coating the NPLs with EDTMP at 80 °C, which agrees with XANES analysis, implying that around 15% of Fe^3+^ ions were in the form of Fe–EDTMP complex. The Ms reduction was lower, around 8 wt%, for the DPA coatings, indicating that NPLs decomposed less than with EDTMP.

Similarly, the formation of a paramagnetic Fe-phosphate shell on phosphated superparamagnetic maghemite nanoparticles was also accompanied by a substantial decrease in the Ms^45^. The Fe-phosphate shell could not form without the dissolution/disintegration of the core nanoparticles. We can conclude that the significant Ms reduction resulted from the modified surface crystal structure of magnetic nanoparticles.

## Conclusions

We studied the interaction of ferrimagnetic BSHF NPLs with phosphonic acids and its effect on the magnetic properties of the NPLs. The phosphonic acids formed stable coatings at the NPLs. However, BSHF NPLs coated with a hydrophilic tetraphosphonic acid, EDTMP, partly decomposed at 80 °C, as was observed with ARM-STEM and inferred from spectroscopic analyses. Accordingly, XANES analysis demonstrated that around 85% of Fe^3+^ ions were incorporated in the BSHF NPLs whereas the remaining 15% of Fe^3+^ ions were in the form of Fe–EDTMP complex. As a consequence, the room-temperature saturation magnetisation decreased by 12–22% more than expected from the EDTMP-coating mass fraction. A direct correlation between the disintegration and the saturation magnetisation reduction was confirmed with the Density Functional Theory, corrected by the Hubbard U parameter (DFT + U) computations. In general, the disintegration accompanied by a considerable reduction in the saturation magnetisation was observed when coating BSHF NPLs with a hydrophilic phosphonic acid having a minimum of two phosphonic groups at different C atoms. When BSHF NPLs were coated with a monophosphonic acid or bisphosphonic acid, disintegration was not observed, and the saturation magnetisation decreased as expected for the specific coating mass fraction.

This study shows that the disintegration of magnetic nanoparticles can be inferred from their room-temperature saturation magnetisation, especially when an insoluble product is formed. The same principle, i.e., measurement of physical properties, could be considered to avoid comprehensive and time-consuming analyses when assessing changes in the surface structure and chemistry of nanomaterials in general.

## Methods

### Materials

Ba(NO_3_)_2_ 99.95%, Fe(NO_3_)_3_ × 9H_2_O 98 + %, Sc(NO_3_)_3_ × xH_2_O 99.9%, NaOH 98%, 1-hexanol, and sodium alendronate trihydrate (Na-AL hydrate) were obtained from Alfa Aesar. Octadecylphosphonic acid (OPA) and HNO_3_ were purchased from Sigma-Aldrich. *N*,*N*,*N*,*N*-ethylenediamine tetra(methylene) phosphonic acid hydrate (EDTMP hydrate) was obtained from abcr GmbH. All chemicals were used as received. Metals' concentration in nitrates was determined with an inductively-coupled plasma optical emission spectrometer (ICP-OES Agilent 720).

BSHF NPLs were synthesised hydrothermally with our optimised method^24^ (Section S1.1 in Supplementary Information). Regardless of the overall chemical composition BaFe_14.9_Sc_0.9_O_x_ (measured with EDXS), the Sc^3+^-substitution for Fe^3+^ does not affect the structure and composition of the surface crystal planes nor their thickness (3.0 or 4.1 nm)^25^.

Phosphonate coatings were obtained with hydrophobic monophosphonic acid OPA, and with EDTMP hydrate and Na-AL hydrate; i.e., a source of hydrophilic EDTMP and AL, respectively (Fig. 1). BSHF NPLs were coated with EDTMP and AL in aqueous suspensions. OPA was coated on the NPLs at an oil–water interface between the solution of OPA in hexanol/toluene mixture and an aqueous suspension of BSHF NPLs, respectively. All coatings were prepared at RT, while the EDTMP and AL coatings were also prepared at 80 °C. Details are given in Section S1.2 in Supplementary Information.

As references for the spectroscopic studies, the Fe^3+^-complexes with EDTMP and OPA were synthesised in the same way as the respective coatings where the surface Fe^3+^ ions of BSHF were substituted with Fe^3+^(*aq*) ions from an aqueous solution of Fe(NO)_3_ (Section S1.3 in Supplementary Information). The aqueous solution was kept at pH 1–2 to prevent salt formation by keeping the EDTMP phosphonic groups fully protonated^46,47^ and to prevent the precipitation of Fe^3+^^48^. The Fe–EDTMP complex precipitated while Fe–OPA complex was soluble in the hexanol/toluene mixture and was dried in a rotavapor for analytical purposes.

### Characterization

Basic characterisation of the as-synthesised and coated BSHF NPLs was performed with TEM (Jeol 2100) at 200 kV coupled with energy dispersive X-ray spectroscopy (EDXS, JED 2300 EDS). NPLs were immobilised on a Cu TEM support grid. Their structure was verified using selected area electron diffraction (SAED), while their size (i.e., diameter) distribution was determined with DigitalMicrograph™ Gatan Inc. software and expressed in equivalent diameters. ARM-STEM was done with C_S_-probe corrected scanning transmission electron microscope (Jeol ARM 200CF) at 80 kV simultaneously using high-annular dark-field (HAADF) and annular bright-field (ABF) detectors at 68–180 and 10–16 mrad collection semi-angles, respectively.

Zeta-potential behaviour vs pH was measured in diluted suspensions (0.1 mg/ml) of the as-synthesised and coated BSHF NPLs using ZetaPALS Zeta Potential Analyzer (Brookhaven, Instruments Corporation) and Litesizer 500 (Anton Paar). The pH was adjusted from neutral to acidic or basic pH with 0.1 or 1 M solutions of HCl or NaOH, respectively.

Static magnetic properties of the dried as-synthesised and coated samples were measured at RT with a vibrating sample magnetometer (VSM, LakeShore 7404). The samples were almost magnetically saturated at the maximum applied field of 1 T. Therefore, we considered the magnetization values measured at 1 T as the saturation magnetisation (Ms). The Ms values are reported with uncertainty determined from the uncertainty of ± 1% in the mass measurement.

TGA of the ligands and dried, as-synthesised and coated, BSHF NPLs were performed with thermal analyser (TGA/DSC 2, Mettler Toledo) coupled with a mass spectrometer (MS, Thermostar300, Vacuum Pfeifer) for the evolved gas analysis. The samples were heated from 40–1100 °C at 20 °C/min in a static air atmosphere. The fractions of bonded ligands were quantified via the decomposition step of the organic part of the ligand during thermal treatment following previously reported evaluation^12^. Decomposition of the ligands occurs in the range from 350 to 600 °C for EDTMP, 430–550 °C for OPA, and 200–500 °C for AL.

FTIR spectroscopy was conducted using PerkinElmer Spectrum 400 spectrometer. The spectra of dried samples were obtained with Universal ATR sampling accessory in the range 4000–650 cm^–1^. The presented ATR-FTIR spectra were ATR-corrected to be comparable with transmittance spectra but without any effect on the band positions. Additional spectra were acquired in DRIFT mode over the range of 4000–400 cm^−1^ with the 4 cm^−1^ resolution. For this purpose, 2 mg of samples were finely ground with 80 mg of KBr.

XANES and EXAFS measurements of the EDTMP and OPA coated BSHF NPLs were performed at P65 beamline of PETRA III at DESY in Hamburg. In addition, uncoated BSHF NPL sample and Fe–EDTMP complex were measured for comparison. The XAS spectra were measured in transmission detection mode. The samples were prepared in the form of homogeneous pellets, pressed from the grounded sample, mixed with the boron nitride (BN) powder to obtain the total absorption thickness (μd) of about 2 above the Fe K-edge. A Si(111) double crystal monochromator was used with an energy resolution of about 1 eV at 7 keV. Higher-order harmonics were effectively eliminated by a flat mirror. The beam size on the sample was 1.5 mm horizontally and 0.2 mm vertically. The intensity of the monochromatic X-ray beam was measured by three consecutive ionisation detectors filled with optimal gas mixtures for a given energy range to provide 15% absorbance in the first ionisation cell filed with a mixture of 15% Ar and 85% N2, 60% absorbance in second ionisation cell filled with Ar, and 80% absorbance in the third ionisation cell filled with Kr. The samples were inserted in the monochromatic X-ray beam between the first and second ionisation cells. The absorption spectra were measured in the energy region from – 150 to + 1000 eV relative to the Fe K-edge (7112 eV) in continuous fast (3 min) scans and re-binned to equidistant energy steps of 0.25 eV in the XANES region and equidistant k steps of 0.05 Å^–1^ in EXAFS region. The exact energy calibration was established with simultaneous absorption measurement on a 5-μm thick Fe foil placed between the second and third ionisation detector. We performed three to five repetitions of the same scan to check the reproducibility and improve the signal-to-noise ratio. The quantitative analysis of XANES and EXAFS spectra was performed with the Demeter (IFEFFIT) program package^49^ in combination with the FEFF6 program code for ab initio calculation of photoelectron scattering paths^50^.

### Computational details

An atomic-level description of the BHF NPLs was performed within the framework of Density Functional Theory, corrected by the Hubbard U parameter (DFT + U)^35^, as implemented in the QuantumEspresso package for electronic structure calculations^51^. We used the Perdew-Burke-Ernzerhof (PBE) exchange–correlation functional^52^, employing a plane-wave basis set with a kinetic energy cutoff of 50 Ry (500 Ry for the charge density cutoff) in combination with ultrasoft pseudopotentials^53^. The Brillouin zone integrations were performed with a 4 × 4 × 1 uniform Monkhorst–Pack grid^54^. The electronic occupations are set according to the Methfessel-Paxton smearing^55^ with a value of 0.001 Ry. Molecular graphics were produced by the XCrysDen graphical package^41^.

Given that BHF bulk is ferrimagnetic, spin polarization was considered, and the starting magnetic moment was set following the two antiferromagnetically aligned Fe^3+^ sublattices (Fe^3+^ ions in lattice sites *2a*, *2b* and *12k* versus *4f1* and *4f2*).

## Supplementary Information

Supplementary Information 1.

Supplementary Information 2.

Supplementary Information 3.

## Data Availability

All data generated or analysed during this study are included in this published article and its Supplementary Information files.
