# Supplementary material for: Saturation magnetisation as an indicator of the disintegration of barium hexaferrite nanoplatelets during the surface functionalisation
Source: Sci Rep. 2023 Jan 19;13:1092. doi: 10.1038/s41598-023-28431-4 (PMC9852462; doi:10.1038/s41598-023-28431-4)
Supplement: Supplementary file 1 — Supplementary Information 1. [file 41598_2023_28431_MOESM1_ESM.pdf]

Supplementary Information

Saturation magnetisation as an indicator of the disintegration of barium hexaferrite nanoplatelets during the surface functionalisation

Darja Lisjak[1*], Iztok Arčon[1,2], Matic Poberžnik[3], Gabriela Herrero-Saboya[3], Ali Tufani[1], Andraž Mavrič[2], Matjaz Valant[2], Patricija Hribar Boštjančič[1,4], Alenka Mertelj[1], Darko Makovec[1], Layla Martin-Samos[3]

[1] Jožef Stefan Institute, SI-1000 Ljubljana, Slovenia

[2] University of Nova Gorica, SI-5000 Nova Gorica, Slovenia

[3] CNR-IOM, Democritos National Simulation Center, Istituto Officina dei Materiali, c/o SiSSA, IT-34136 Trieste, Italy

[4] Jožef Stefan International Postgraduate School, SI-1000 Ljubljana, Slovenia

*corresponding author darja.lisjak@ijs.si

S1. Materials details

S1.1 Barium hexaferrite nanoplatelets

Barium hexaferrite nanopletelets, partly substituted with Sc^{3+} (BSHF NPLs), were synthesized hydrothermally at 245 °C as previously in ref.[1]. The BSHF NPLs were dispersed in a nitric acid solution (14.3 M, pH 2.0–2.2) and, as such, used in the coating processes. When needed for specific characterization, they were dried at 80 °C.

S1.2 Coating the BSHF NPLs

BSHF NPLs were coated as previously[2]. Ethylenediaminetetra(methylene phosphonic acid) (EDTMP) and alendronic acid (AL) (Figure 1) were obtained with a maximum of one deprotonated –OH group in each of the phosphonic groups[3–5] by dissolving respective reagents (EDTMP hydrate and sodium alendronate trihydrate) at the coating temperature at pH 2–3 and <6, respectively. The pH was adjusted with the HNO_3 solution. The BSHF NPLs suspension was admixed to the phosphonic-acid solution and stirred with a glass stirrer for 3 h at room temperature (RT) or 80 °C. The nominal fractions of phosphonic acids corresponded to ~8 EDTMP/nm^2 and ~10 AL/$nm^{2,}$ and the NPLs concentration during the reaction was set to 0.1 mg/ml. The coated NPLs were washed with water several times with intermediate centrifugation. The samples were named BSHF@EDTMP and BSHF@AL.

BSHF NPLs were coated with octadecylphosphonic acid (OPA; Figure 1) at a phase boundary. The OPA solution in toluene:1-hexanol (4:1) was stirred with the stable aqueous suspension of NPLs for 2 h at RT. The fraction of phosphonic acid corresponded to ~17 OPA/nm^{2}, and the reaction concentrations of NPLs was 0.1 mg/ml. During the reaction, the coated BSHF NPLs transferred to the toluene phase and were separated from the aqueous phase in a separating funnel, and subsequently washed with the reaction solvents several times with intermediate centrifugation. The samples were named BSHF@OPA.

S1.3 Synthesis of Fe–phosphonate complexes

Fe-EDTMP and Fe-OPA complexes were synthesized in the same way as respective coatings.
For the Fe-EDTMP, $Fe(NO_3)_3$ was dissolved in water (16.5 mM) and reacted with the EDTMP solution at pH 1–2 for 3 h at 80 °C. The molar ratio of Fe/EDTMP was 1:1, meaning that the Fe/P molar ratio was 1:4. The precipitous product was washed in the same way as BSHF@EDTMP.
For the Fe-OPA, the aqueous solution of $Fe(NO_3)_3$ (30 mM) was mixed with a solution of OPA in a toluene/hexanol mixture for 2 h at RT with the Fe/OPA molar ratio 4:1. During the mixing, the oily phase turned yellow, indicating the transfer of Fe^{3+} ions from the aqueous to the oil phase. This was an indication that Fe^{3+} ions interacted with OPA. The product did not precipitate. We separated the oily phase in a separating funnel and dried it in a rotavapor for the spectroscopic analyses.

S2. BHF NPL bulk termination slab models

The BHF NPLs present a relatively large surface area to thickness ratio. A single NPL was approximated as a slab subject to periodic boundary conditions (PBC) for the basal directions and a vacuum layer >15 Å along the main axis. The slab is cut by two basal planes from the crystal, ensuring the same bulk termination for both surfaces. An R block of BHF bulk is taken as the central structural unit, and the basal planes slice the bulk along atomic layers guaranteeing the same thickness above and below the referent R block. In this manner, slabs with different thicknesses are obtained, starting from a minimal width given by the *2a* lattice sites. Therefore, a slab with *12k* termination is constituted by the referent R block and 2S blocks. A 4×4×1 uniform Monkhorst-Pack grid[6] was employed for all considered slabs.

S3. Supporting results

Figure S1

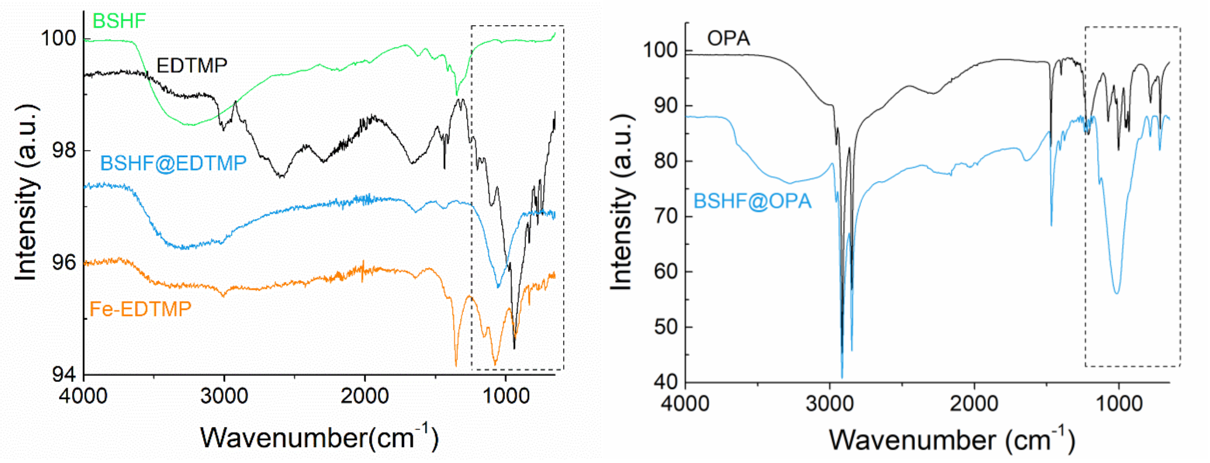

Figure S1. ATR-FTIR spectra of the core and coated BSHF NPLs, pure ligands, and the Fe-EDTMP complex. BSHF@EDTMP and Fe–EDTMP samples were prepared at 80 °C. Characteristic phosphonic-group bands are within the dashed rectangular.

Fe K-edge EXAFS results, Figure S2 and Tables S1–S4

Average local structure of Fe cations in the EDTMP- and OPA-coated BSHF NPLs and in reference BHF NLPs and Fe-EDTMP complex is obtained by EXAFS analysis. Structural parameters of the average Fe local neighbourhood (type and an average number of neighbors, the radii and Debye-Waller factor of neighbor shells) are quantitatively resolved from the EXAFS spectra by comparing the measured EXAFS signal with model signal, constructed *ab initio* with the FEFF6 program code[7,8]. The atomic species of neighbors are identified in the fit by their specific scattering factor and phase shift.

The FEFF model for Fe cations in the BSHF NLPs is based on the hexagonal crystal structure of BHF with space group *P 63/mmc* with the lattice constants $a = b = 5.800$ Å, $c = 23.180$ Å [9,10]. To describe the average local Fe neighbourhood in the NPLs, where Fe occupies five distinct sites in the BHF crystal structure, a simplified EXAFS model is constructed, built with just one oxygen neighbor shell, two Fe and three Ba neighbor shells, as in previous EXAFS analysis of similar BHF structure[11]. The FEFF model comprised six single scattering paths in the R range up to 3.6 Å, with eleven variable parameters: coordination shell number (N), distance (R) and Debye-Waller factors (σ^2) of each scattering path, and a shift of energy origin of the photoelectron ΔE_o, common to all scattering paths. The amplitude reduction factor S_0^2 was kept fixed at the value of 0.75. To stabilize the fit of the very weak contributions of Ba neighbors, the Debye-Waller factors of their scattering paths were constrained to a common value. The model is tested on the EXAFS spectrum measured on the reference core BSHF NPLs. A very good EXAFS fit is obtained in the *k* range of 3 – 13 Å^{-1} and the *R*-range of 1.2 – 3.6 Å (Figure S2). The best fit structural parameters are given in Table S1.

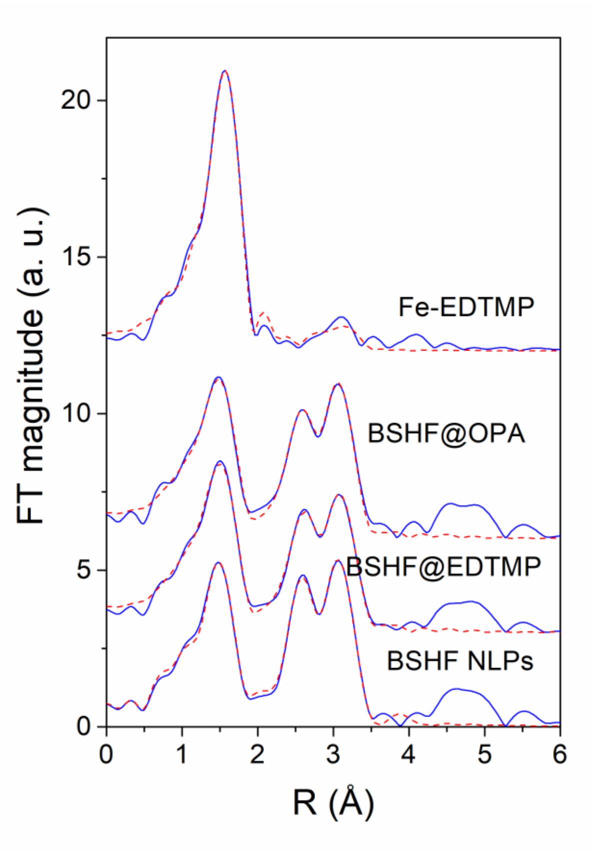

Figure S2. Fourier transform magnitudes of the k^3-weighted Fe K-edge EXAFS spectra of the BSHF@EDTMP and BSHF@EDTMP NLP samples and reference spectra of the core BSHF NLPs and Fe–EDTMP complex, calculated in the k range of 3 Å^{-1} to 13 Å^{-1} (solid blue line – experiment, red dashed line – best fit EXAFS model in the R range of 1.2–3.6 Å). The spectra are shifted vertically for clarity. BSHF@EDTMP and Fe–EDTMP samples were prepared at 80 °C.

For the spectrum of Fe-EDTMP reference sample, which shows significantly different Fe local structure, a separate FEFF model was constructed, with octahedral coordination of 6 oxygen atoms in the nearest Fe coordination shell at a distance of 2.0 Å, and two oxygen and one P atoms in the second coordination shell in the R range between 3.0 Å to 3.5 Å, which can be expected for Fe^{3+} cations attached to the phosphonic group in the Fe-EDTMP complex, forming Fe-O-P bridges. The FEFF model included three single scattering paths, with 8 variable parameters: coordination shell distances (R), and Debye-Waller factors (σ^2) of each scattering path, and the shift of energy origin of the photoelectron ΔE_o, common to all scattering paths. The coordination numbers (N) of the second coordination shell neighbors, which produce a weak EXAFS signal, were not varied. The amplitude reduction factor S_0^2 was kept fixed at the value of 0.75. The FEFF model describes very well the EXAFS spectrum of the Fe-EDTMP reference sample in the k range of 3 – 13 Å^{-1} and the R-range of 1.0 – 3.5 Å (Fig S2). The best fit structural parameters are given in Table S2.

The EXAFS spectra of the EDTMP or OPA coated BHF NPL samples can be described entirely with the FEFF model for Fe cations in the BHF NLPs. A very good EXAFS fit is obtained in the k range of 3 – 13 Å^{-1} and the R-range of 1.2 – 3.6 Å (Figure S2). The best fit structural parameters are given in Tables S3 and S4. Fe EXAFS results show that the average Fe local structure of both samples is practically the same as that in the BSHF NPLs reference sample. We also tested the possible contribution of the Fe cations bound to the organic matrix,

indicated by XANES analysis. The scattering paths from the FEFF model used to describe the spectrum of the Fe-EDTMP reference sample were added. However, the contribution of this phase to the total EXAFS signal is below the detection limit. Nevertheless, we have to point out that the presence of the small amount of Fe cations bound to the organic matrix, as demonstrated by XANES analysis, is not excluded by EXAFS fits.

Table S1. Parameters of the nearest coordination shells around Fe cations in the reference BSHF NLP sample: average number of neighbor atoms (N), distance (R), and Debye-Waller factor (σ^2). Uncertainty of the last digit is given in parentheses. The best fit is obtained with the amplitude reduction factor S_0^2=0.80(5) and the shift of the energy origin ΔE_o = –2(1) eV. The R-factor (quality of fit parameter) is listed in the last column.

Fe neigh.	*N*	*R* [Å]	σ^2 [Å^2]	*R*-factor
**BSHF NLPs**				
O	4.7(5)	1.934(3)	0.008(2)	0.0029
Fe	3(1)	2.97(1)	0.011(2)	
Ba	0.2(1)	3.46(1)	0.008(3)	
Fe	6(1)	3.49(2)	0.009(2)	
Ba	0.4(1)	3.66(1)	0.008(2)	
Ba	0.4(1)	3.74(1)	0.008(2)	

Table S2. Parameters of the nearest coordination shells around Fe cations in the reference Fe-EDTMP complex: average number of neighbor atoms (N), distance (R), and Debye-Waller factor (σ^2). Uncertainty of the last digit is given in parentheses. The best fit is obtained with the amplitude reduction factor S_0^2=0.80(5) and the shift of the energy origin ΔE_o = –2(1) eV. The R-factor (quality of fit parameter) is listed in the last column.

Fe neigh.	*N*	*R* [Å]	σ^2 [Å^2]	*R*-factor
**Fe-EDTMP complex**				
O	6.0(3)	1.98(1)	0.006(1)	0.0052
O	2	3.08(2)	0.006(1)	
P	1	3.52(2)	0.005(2)	

Table S3. Parameters of the nearest coordination shells around Fe cations in the BSHF@EDTMP NPL sample: average number of neighbor atoms (N), distance (R), and Debye-Waller factor (σ^2). Uncertainty of the last digit is given in parentheses. The best fit is obtained with the amplitude reduction factor S_0^2=0.80(5) and the shift of the energy origin ΔE_o = –2(1) eV. The R-factor (quality of fit parameter) is listed in the last column.

Fe neigh.	*N*	*R* [Å]	σ^2 [Å^2]	*R*-factor
**BSHF@EDTMP NPLs**				
O	5.1(5)	1.944(3)	0.009(2)	0.0028
Fe	4(1)	2.98(1)	0.013(2)	
Ba	0.2(1)	3.46(1)	0.008(3)	
Fe	4(1)	3.51(2)	0.008(2)	
Ba	0.4(1)	3.66(2)	0.008(2)	
Ba	0.4(1)	3.74(2)	0.008(2)	

Table S4. Parameters of the nearest coordination shells around Fe cations in the BSHF@OPA NLP sample: average number of neighbor atoms (N), distance (R), and Debye-Waller factor (σ^2). Uncertainty of the last digit is given in parentheses. The best fit is obtained with the amplitude reduction factor S_0^2=0.80(5) and the shift of the energy origin ΔE_o = –2(1) eV. The R-factor (quality of fit parameter) is listed in the last column.

Fe neigh.	*N*	*R* [Å]	σ^2 [Å^2]	*R*-factor
**BSHF@OPA NLPs**				
O	5.0(4)	1.934(3)	0.009(2)	0.0020
Fe	4(1)	2.96(2)	0.012(2)	
Ba	0.2(1)	3.46(1)	0.008(3)	
Fe	5(1)	3.50(2)	0.009(2)	
Ba	0.4(1)	3.66(2)	0.008(2)	
Ba	0.4(1)	3.74(2)	0.008(2)	

Figures S3 and S4

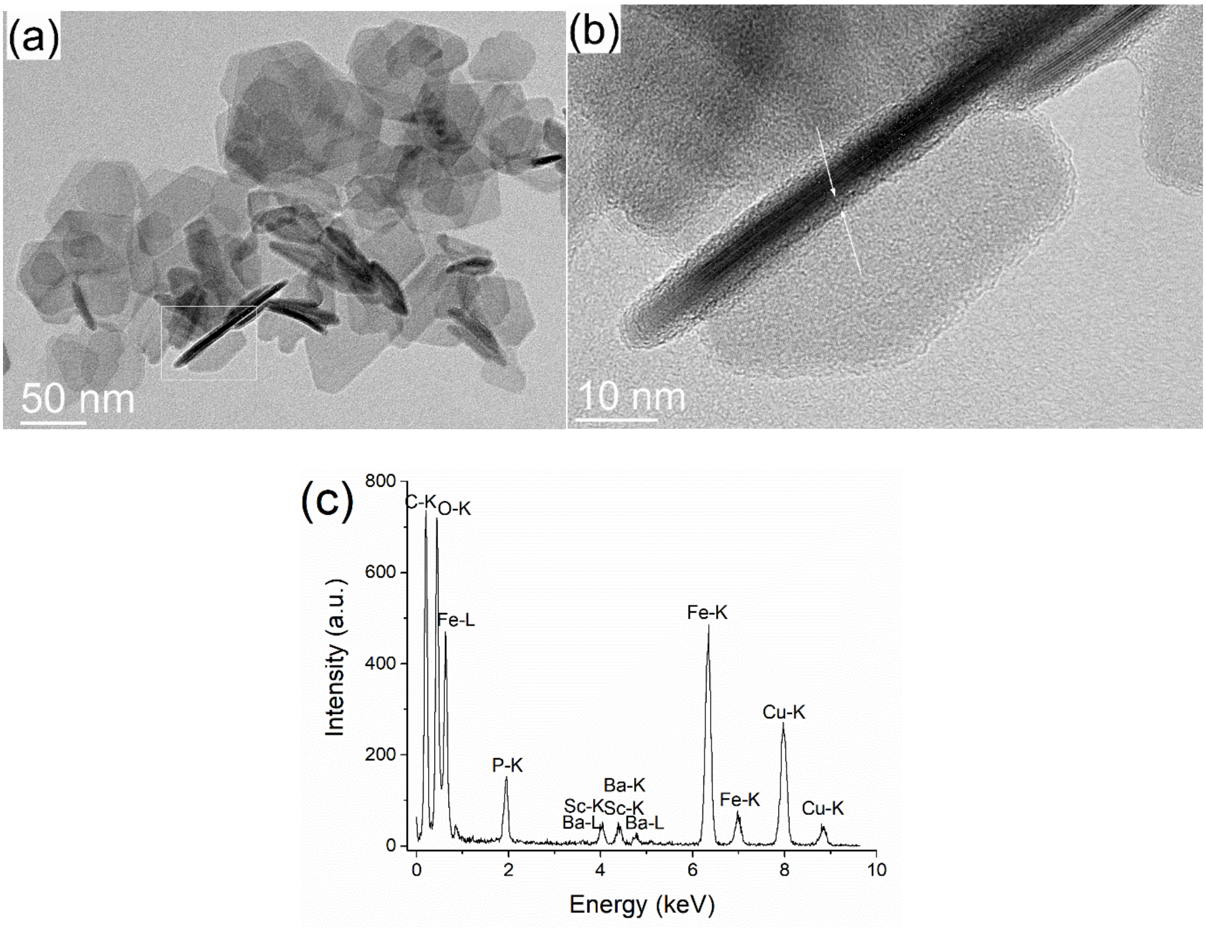

Figure S3. TEM image of the BSHF@AL NPLs (a) with an area within the white square taken at higher magnification (b) where arrows point at an amorphous surface layer. The corresponding EDXS spectrum is shown in panel (c).

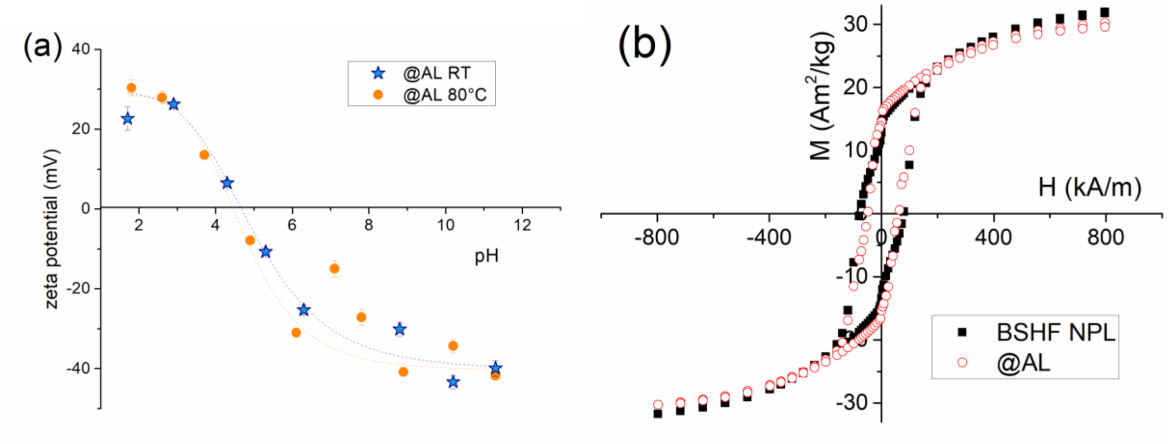

Figure S4. Zeta-potential behavior of the aqueous suspension of the BSHF@AL NPLs coated at RT and 80 °C (a), and magnetic hysteresis of the core and BSHF@AL NPLs coated at 80 °C (b). The fitting lines in panel (a) serve only for a clearer presentation.

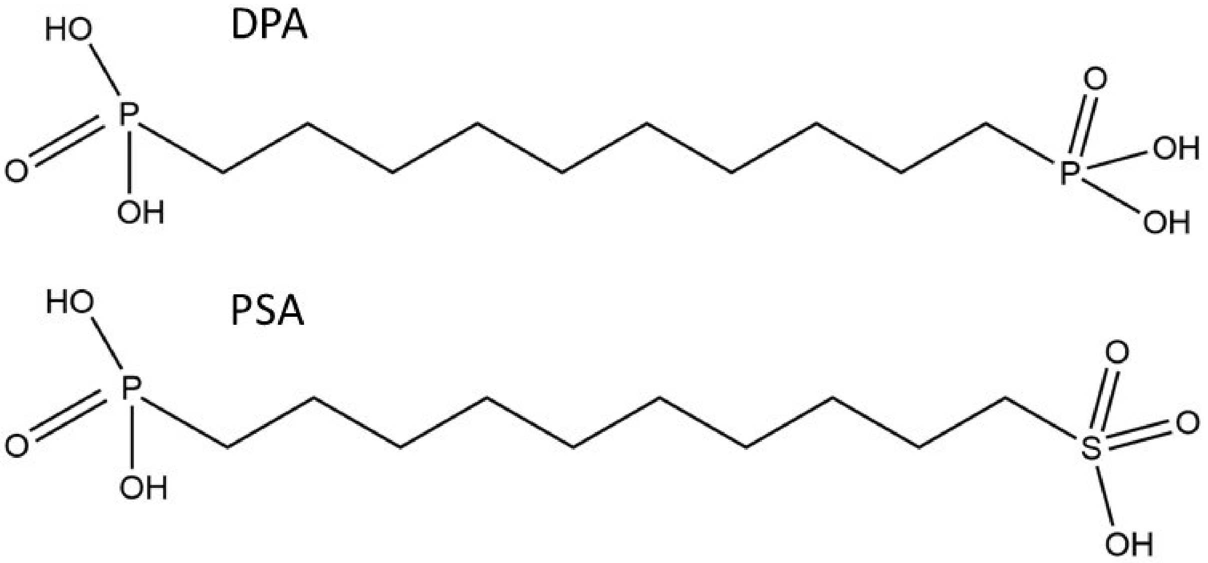

Figure S5. Hydrophilic phosphonic acids considered in our comparison in Section 4.4: (12-phosphono)dodecyl phosphonic acid (DPA) – partly soluble in water, and (12-phosphono)dodecyl sulfonic acid (PSA) – fully soluble in water.

References

1. Lisjak, D. & Drofenik, M. Chemical substitution-an alternative strategy for controlling the particle size of barium ferrite. *Cryst. Growth Des.* **12**, 5174–5179 (2012).
2. Lisjak, D. *et al.* Formation of Fe(III)-phosphonate Coatings on Barium Hexaferrite Nanoplatelets for Porous Nanomagnets. *ACS Omega* **5**, 14086–14095 (2020).
3. Rizzikala, E. N. & Zaki, M. T. Metal chelates of phosphonate-coating ligands-I", Stability of some N’,N’,N’N’-ethylenediaminetetra(methylendphosphonic) acid metal chelates. *Talanta* **26**, 507–510 (1979).
4. Westerback, S., Rajan, K. S. & Martell, A. E. New Multidentate Ligands. III. Amino Acids Containing Methylenephosphonate Groups. *J. Am. Chem. Soc.* **87**, 2567–2572 (1965).
5. Ke, J. *et al.* Determination of pKa values of alendronate sodium in aqueous solution by piecewise linear regression based on acid-base potentiometric titration. *J. Pharm. Anal.* **6**, 404–409 (2016).
6. Monkhorst, H. J. & Pack, J. D. Special points for Brillouin-zone integrations. *Phys. Rev. B* **13**, 5188–5192 (1976).
7. Ravel, B. & Newville, M. ATHENA, ARTEMIS, HEPHAESTUS: data analysis for X-ray absorption spectroscopy using IFEFFIT. *J. Synchrotron Radiat.* **12**, 537–541 (2005).
8. Rehr, J. J., Albers, R. C. & Zabinsky, S. I. High-order multiple-scattering calculations of x-ray-absorption fine structure. *Phys. Rev. Lett.* **69**, 3397–3400 (1992).
9. J. J. Went, G. W. Rathenau, E. W. Gorter, G. W. van O. Ferroxdure a class of new permanent magnet materials. *Philips Tech. Rev.* **13**, 194–208 (1952).
10. Pollert, E. Crystal chemistry of magnetic oxides part 2: Hexagonal ferrites. *Prog. Cryst. Growth Charact.* **11**, 155–205 (1985).
11. Makovec, D. *et al.* Structural properties of ultrafine Ba-hexaferrite nanoparticles. *J. Solid State Chem.* **196**, 63–71 (2012).
